# Detection of *Echinococcus granulosus sensu lato* in Environmental Samples from Ibadan, Oyo State, South West Nigeria

**DOI:** 10.3390/vetsci9120679

**Published:** 2022-12-07

**Authors:** Emmanuel Jolaoluwa Awosanya, Adeola Olagbaju, Angela Peruzzu, Gabriella Masu, Giovanna Masala, Piero Bonelli

**Affiliations:** 1 Department of Veterinary Public Health and Preventive Medicine, University of Ibadan, Ibadan 200005, Nigeria; 2 National Reference Laboratory for Cystic Echinococcosis (CeNRE), WOAH Reference Laboratory for Echinococcosis, Istituto Zooprofilattico Sperimentale della Sardegna (IZS), Via Vienna 2, 07100 Sassari, Italy

**Keywords:** *Echinococcus granulosus sensu lato*, taeniid eggs, environmental contamination, Nigeria

## Abstract

**Simple Summary:**

The presence of parasite eggs in the environment represents an alarming threat for human health. In particular, the accidental ingestion of tapeworm eggs can cause severe and disabling illness in humans. Cystic Echinococcosis (CE), caused by infection from the tapeworm *Echinococcus granulosus*, gives rise to great concern for its medical and economic burden. CE transmission to humans mostly occurs through the consumption of water or food contaminated by feces of infected dogs. In the present study, we collected soil, fecal and water samples in the city of Ibadan, Oyo State, Nigeria, to detect parasite contamination in the environment. We evidenced that the urban and semi-urban areas of Ibadan are highly contaminated with *E. granulosus s.l.* eggs, emphasising the need for appropriate measures to control CE. These findings suggest that prevention strategies should consider the control of stray dogs, the establishment of deworming programs and the promotion of public education.

**Abstract:**

Environmental contamination with parasite eggs poses a serious risk to public health. This study aimed to assess the presence of taeniid eggs and, in particular, *E. granulosus s.l.*, in environmental samples in the city of Ibadan, South West Nigeria. To this purpose, soil (*n* = 200), fecal (*n* = 200) and water samples (*n* = 50) were examined by microscopic observation and the multiplex PCR method. The influence of specific environmental factors on *E. granulosus s.l.* egg dispersion was also evaluated. Taeniid eggs were microscopically found in 11.5%, 25.5% and 8.0% of soil, fecal and water samples, respectively. PCR analyses evidenced the presence of *E. granulosus s.l.* in 8.0%, 24.0% and 2.0% of soil, fecal and water samples, respectively. The proximity to slaughterhouses, the level of urbanisation and the local government area of belonging did not seem to affect *E. granulosus s.l.* egg dissemination patterns. Our results have clearly demonstrated that both urban and semi-urban areas of the city of Ibadan in Nigeria are highly contaminated by taeniid eggs and we recommend the adoption of appropriate measures to control *E. granulosus s.l.*

## 1. Introduction

The larval form of *Echinococcus granulosus sensu lato* (*s.l.*) is the cause of cystic echinococcosis (CE) in humans and animals, a zoonotic and neglected tropical disease that is listed second among foodborne illnesses by the World Health Organisation (WHO) [1]. Five different species of *E. granulosus s.l.* have been identified: *Echinococcus granulosus sensu stricto (s.s.)*, *Echinococcus ortleppi*, *Echinococcus equinus*, *Echinococcus canadensis* and *Echinococcus felidis* [2]. Among these species, *E. granulosus s.s.* causes great concern as it is responsible for the vast majority of human CE cases worldwide. *E. canadensis* contributes to a lesser extent of the global burden of human CE and can become predominant in particular regions [3].

The life cycle of *E. granulosus s.l.* involves definitive (carnivores) and intermediate hosts (ungulates). The adult worms live in the small intestine of the definitive hosts and produce eggs contained in mature proglottids that are excreted within feces. Intermediate hosts become infected, usually at pasture, by ingesting mature proglottids. Oncospheres released by the eggs develop into metacestode and cause the formation of CE cysts in the internal organs. The completion of the life cycle occurs when the definitive hosts feed on the infected organs of the intermediate hosts [4]. Humans can acquire an infection that acts as dead-end host, as they do not contribute to the parasite’s perpetuation. Domestic dogs have a prominent role in the transmission of *E. granulosus* infection to humans, either directly or indirectly, through the contamination of soil, water or vegetables [5]. It has been demonstrated that taeniid eggs may remain viable in the natural environment for long periods of time [6], and in particular, *E. granulosus* eggs maintained their viability and infectiveness for up to 41 months [7]. The large number of *E. granulosus* proglottids shed in feces by dogs and the high survival and dispersal capacity of eggs outdoors point out the importance of the environmental contamination for public health [4,5,6].

CE is highly endemic in Sub-Saharan Africa, but its true burden is still unknown. In Nigeria, most studies have focused on establishing the prevalence of *Echinococcus* infection in the definitive host [8,9,10,11,12] and CE among the intermediate hosts [13,14,15], including humans [12,16]. Although the human health challenge represented by CE is widely recognised, to our knowledge, the *E. granulosus* infection risk associated with environmental contamination in Nigeria has never been investigated. This study, therefore, aimed to estimate the presence of taeniid and *E. granulosus s.l.* eggs in soil, dog feces and water samples collected from the urban and semi-urban areas in the city of Ibadan in Nigeria.

## 2. Materials and Methods

### 2.1. Study Design

**Figure 1:**
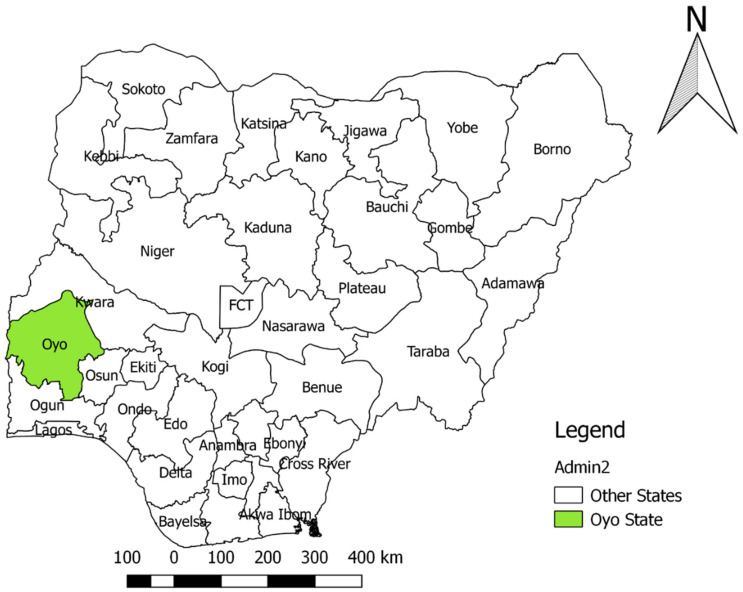
Map of Nigeria highlighting Oyo State in green.

We have carried out a cross sectional study between March and September 2018. The study area was Ibadan, Oyo State, South West Nigeria (Figure 1).

Ibadan is located in South West Nigeria, 128 km inland northeast of Lagos and 120 km east of the Nigerian international border with the Republic of Benin. It sits completely within the tropical forest zone, close to the boundary between the forest and the derived savannahs. The city ranges in elevation between 150 m, in the valley area, and 275 m above sea level on the major north-south ridge, which crosses the central part of the city. The city covers a total area of 3080 km^2^, being the largest in Nigeria [17].

There are eleven Local Governments Areas in the Ibadan Metropolitan area, consisting of five urban local governments (Ibadan North, Ibadan North-East, Ibadan North-West, Ibadan South-East, and Ibadan South-West) and six semi-urban local governments (Akinyele, Egbeda, Ido, Lagelu, OnaAra, Oluyole) [18]. The human population of Ibadan is estimated to be 3,649,000 [19]. The study locations covered three of the 11 Local Government Areas in the Ibadan metropolitan areas: Ibadan North, Ibadan South-West and Akinyele Local Government Areas.

### 2.2. Sample Size and Sampling

A total of 200 soil, 200 fecal and 50 water samples were collected. We calculated the sample size (*n*) using the formula [20]

*n* = (Z^2^ p (1 − p))/d^2^,

where Z is the reliability coefficient of 1.96 at 95% confidence level; p is the prevalence of taeniid eggs in dogs at 4.86% [21]; d is the precision at 5%.

**Figure 2:**
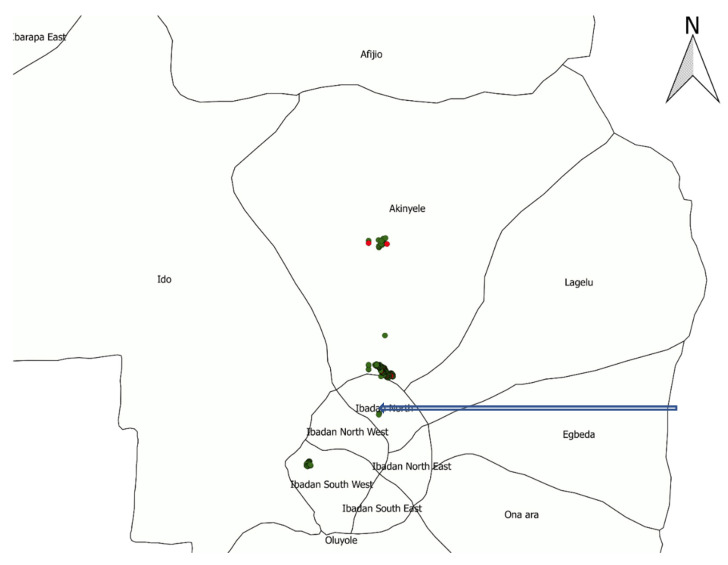
Samples collection points across the three Local Government Areas covered in Ibadan Metropolitan area, Oyo State. Dots represent *Echinococcus granulosus* positive (red) and negative (green) results. The arrow indicates the position of the abattoir.

The environmental samples were obtained at increasing distances from an abattoir in the city centre; 10, 15, 20, 25, 35 and 50 dog feces and soil samples were, respectively, collected at 5, 10, 20, 40, 100, 200, 500 m and 10 km of distance (Figure 2).

Approximately 5 g of dog feces were obtained from the environment by using polythene covers and stored at −80 °C. Only freshly voided feces, still soft and moist, were collected. It was not possible to attribute a correspondence between the fecal samples found and known animals. As the occurrence of the feces belonging to the same dog cannot be excluded, the number of samples might represent a lower number of dogs. Soil samples were collected from a depth of 3–5 cm in areas usually frequented by dogs [22].

The water samples (10 mL) were collected into sample bottles, obtained from available open water sources and drainages and stored at 4 °C until use.

### 2.3. Microscopic Analyses

The soil samples were prepared using the O’Lorcain method of flotation, as already described [22]. Briefly, 25–30 mL of 1% Tween 80 was added to a sieved soil sample (20 g) in a conical flask. This was vortexed and sieved into glass tubes. The filtrate was centrifuged at 1500 rpm for 5 min. The sediment was washed twice with distilled water and resuspended in a saturated NaNO_3_ solution (1.35 s.p.). Coverslips were placed on top of the tubes, which were centrifuged at 4000 rpm for 15 min.

A sedimentation technique was used for the fecal samples, as previously described [11]. The water samples were prepared as follows: approximately 5 mL of silt sediment was collected from each sample and thoroughly mixed with 35 mL of Sheather’s solution (1.30 s.p.). The mixture was divided into two 20 mL glass tubes. Both were filled with additional Sheather’s solution and coverslips were placed on top. The mixture was centrifuged at 2000 rpm for 10 min.

The microscopic examination of the soil, feces and water samples was performed to detect taeniid eggs by two different operators. Firstly, the slides were observed, at the optic microscope at 4×, in an ordered pattern. Parasitic eggs were morphologically identified at 10× and 40× magnification. The procedure was repeated until approximately 25% of the sediment in any given sample was used. Samples were considered positive if at least one taeniid egg was detected by both operators.

### 2.4. Multiplex Polymerase Chain Reaction Analyses

The detected taeniid eggs were further identified using a multiplex *PCR* [23] at the WOAH Reference Laboratory for Echinococcosis in Italy. The nad1 gene for *Echinococcus multilocularis*, rrnS gene for *E. granulosus s.l.* and rrnS gene for *Taenia* spp. were targeted for amplification. The total reaction mix volume was 25 µL: Master Mix (Quanti-Tect Probe PCR Master Mix—QIAGEN^®^) (12.5 µL); Primer Cest 1: 2 pmol/µL (0.5 µL); Primer Cest 2: 2 pmol/µL (0.5 µL); Primer Cest 3: 2 pmol/µL (0.5 µL); Primer Cest 4: 2 pmol/µL (0.5 µL); Primer Cest 5: 16 pmol/µL (0.5 µL); water (5 µL); and 5 µL of each DNA samples were added (5 ng DNA per tube). Reference samples of *Echinococcus granulosus* DNA from the WOAH Reference laboratory 10 ng (5 µL) were used as the positive control, while 5 µL of sterile water was used as the negative control.

Agarose gel electrophoresis was performed on precast gels (E-Gel™ EX Agarose Gels, 2% Agarose, Thermo Fisher, Waltham, MA, USA) using the E-Gel™ Power Snap Electrophoresis System (Thermo Fisher). The DNA ladder (Marker VIII, Sigma-Aldrich, Saint Louis, MO, USA) was loaded onto the agarose gel in order to determine the size of the PCR products.

### 2.5. Data Analysis

All of the data were entered into Microsoft Excel^®^ 2010 and analysed using Epi Info^®^ version 7.2.4.0. Descriptive statistics, such as frequencies, proportions and percentages, were determined. We also determined the odds ratios and the 95% confidence intervals in order to assess the association between the outcome variable and the independent variables, such as the distances from the abattoir, level of urbanisation, and Local Government Areas. The outcome variable in the bivariate analyses was the proportion of *E. granulosus s.l.* positive samples. A Fisher’s exact test, at 5% level of significance, was performed.

## 3. Results

The microscopic examination of the environmental samples revealed the presence of taeniid eggs in 23 out of 200 soil samples (11.5%, 95% Confidence Interval, CI: 7.7–16.7%); 51 out of 200 dog faecal samples (25.5%, 95% CI: 19.8–32.0%); and four out of the 50 water samples (8.0%, 95% CI: 2.6–19.4%).

Multiplex PCR analyses, performed on the DNA extracted from the environmental samples, evidenced the presence of *E. granulosus s.l.* in 8.0% (95% CI: 4.9–12.7%) and 24.0% (95% CI: 18.6–30.4%) of soil and fecal samples, respectively (Table 1 and Table 2).

Of the 50 water samples examined, the presence of *E. granulosus s.l.* was evidenced in one sample (2.0%, 95% CI: 0.0–11.5%). Moreover, 14 soil (7%, 95% CI: 4.1–11.5%) and 32 fecal samples (16.0%, 95% CI: 11.5–21.8%), out of the 200 samples tested, as well as one out of the 50 water samples (2.0%, 95% CI: 0.0–11.5%), were found positive for *Taenia* spp.

*E. granulosus s.l.* and *Taenia* spp. were simultaneously detected in 10 samples (5.0%, 95% CI: 2.6–9.1%) and 32 samples (16.0%, 95% CI: 11.5–21.8%) out of the 200 soil and faecal samples, respectively. All of the samples were found to be negative for *E. multilocularis*.

The presence of *E. granulosus s.l.* in the soil samples was found to a variable extent, depending on the increasing distance (5 m, 10 m,20 m, 40 m, 100 m, 200 m, 500 m, 10 km) from the abattoir located in the city centre. No positive sample was detected at the 5 m distance. The percent positivity found at 100 m (24.0%) was significantly higher than that found at 200 m (2.9%) and 10 km (4.0%) (Table 1). Other environmental categorisations, such as the different level of urbanisation and the Local Government Areas, were not significantly associated with the presence of *E. granulosus s. l.* (Table 1).

As shown in Table 2, the presence of *Echinococcus granulosus s.l.* in the fecal samples was evidenced at 5 m, 10 m, 20 m, 40 m, 100 m, 200 m, 500 m and 10 km from the abattoir. The percent positivity found at 500 m (5.7%) was significantly lower than that found at 10 m (50.0%) and 10 km (26%) (Table 2). In addition, the different levels of urbanisation and the Local Government Areas were not significantly associated with the presence of *E. granulosus s.l.* in the fecal samples (Table 2).

## 4. Discussion

In this work, we aimed to evaluate the environmental contamination by taeniid eggs, and in particular *E. granulosus s.l.*, in the city of Ibadan in South West Nigeria. To this end, soil, dog feces and water samples were collected and analysed by both traditional parasitological and molecular methods. Our results contribute to the evaluation of the environmental contamination caused by taeniid eggs in Nigeria. This information is of crucial importance because of the zoonotic risk determined by taeniids, and especially by *E. granulosus s.l.* eggs, dispersed by infected dogs. Furthermore, the persistence of viable eggs to external climatic conditions [6,24] raises concerns for human exposure, either directly or indirectly, through contaminated food and water [5].

Microscopic examination evidenced substantial levels of taeniid egg contamination in soil and water, concurrently, with higher values found in dogs feces. A multiplex PCR [23] was also performed for the simultaneous detection of *Taenia* spp. and *Echinococcus* in the environmental samples. The higher sensitivity of PCR for taeniid egg detection allowed us to confirm the results obtained by microscopic examination, the latter being easily affected by biases inherent to the visual assessment of images. As expected, the multiplex PCR results for *E. multilocularis* were negative in all of the environmental samples tested. More interestingly, we evidenced the presence of *E. granulosus s.l.* in soil, fecal and water samples. To our knowledge, this is the first report describing the environmental contamination by *E. granulosus s.l.* in Nigeria using molecular tools.

In Nigeria, the exact picture of *E. granulousus* infection is unclear, as investigations have remained scarce in the last few years. A recent meta-analysis of data published over 50 years estimated that Northern Africa displayed the highest prevalence of *E. granulosus* in the African continent, followed by Eastern Africa [12].

Ohiolei et al. [25] reviewed the CE reports in Nigeria, pointing out that the real prevalence in animals and humans was still unknown in many areas of the country and that proper information on environmental contamination is lacking. In Oyo State, high prevalences of *Echinococcus* taeniasis and CE were respectively recorded in definitive [10] and intermediate hosts [13]. Our previous work evidenced a 5.5% prevalence of *E. granulosus s.l.* [11] in owned dogs in Lagos State in Nigeria, which may imply even higher values in different population groups not receiving the same health care as companion animals. As a matter of fact, in this study, we recorded an almost 5-fold higher presence of *E. granulosus s.l.* (24%) in stray dogs compared to owned dogs. The percentage of *E. granulosus s.l.* infection in the dog fecal samples collected from urban and periurban areas were similar to those observed in Tunisia (25%), while those in China were slightly higher (33%) [26,27]. On the contrary, a lower presence of *E. granulosus* was found in Europe (2.2%), Buthan (3.7%) and Chile (2.1%) [28,29,30].

Our findings evidenced the presence of *E. granulosus* both in soil (8%) and water (2%) samples, highlighting the risks for human health related to environment contamination. In particular, fresh vegetables produced in contaminated environments represent a potential source of human infection if consumed raw or without proper washing. Several reports evidenced the presence of taeniid eggs in various types of vegetables collected from local markets in Nigeria, with positive percentages ranging between 2% and 18%. The detection of parasite eggs in vegetables had been correlated with direct contact of the plant with the contaminated soil, as well as with the use of contaminated water for irrigation or post-harvesting washing [31,32,33]. Water had already been associated as a possible route of human infection with *E. granulosus* [34,35], *E. multilocularis* [28] and other tapeworms belonging to the Taeniidae family [36]. Furthermore, stray dogs also play a crucial role on CE transmission in urban areas of endemic regions; in particular, children are reported to be at high risk of infection due to soil contamination in parks and playgrounds [37,38]. There are few studies specifically examining the presence of *E. granulosus* in soil and water; however, our findings evidenced higher levels of environmental contamination than those obtained in rural homesteads in Kazakhstan and in educational farms in Italy [39,40].

The present work also aimed to evaluate the role of different environmental factors (proximity to a slaughterhouse, level of urbanisation, Local Government Area of belonging) in the dissemination of *E. granulosus s.l.* eggs. To this end, soil and fecal samples were collected at increasing distances from a slaughterhouse, in urban and semi-urban contexts and in different Local Government Areas. Our results suggested that soil contamination by *E. granulosus s.l.* appeared to decrease as the distance from the slaughterhouse increased, considering the significant higher presence of the parasite found at 100 m respect to 200 m and 10 km. These results are in contrast with those observed for dog fecal samples, the contamination rate of which was not related to the distance from the slaughterhouse. This is also in agreement with the findings of Chaâbane-Banaoues et al., in Tunisia [26], which reported no association between the dissemination of *E. granulosus s.l.* eggs and the presence of abattoirs in the proximity. The presence of roaming dogs in the slaughterhouse or in the immediate surroundings may imply easy access to infected offal by dogs, resulting in *E. granulosus* cycle perpetration and of the increase in environmental contamination risk [12].

As demonstrated by Sánchez et al. [24], the dispersion of *E. granulosus* eggs and environment contamination seem to be related to the infection burden, the behaviour of dogs (dog movements and habits), the meteorological conditions (wind direction) and the landscape characteristics (presence of vegetation and water). All of these factors, particularly those regarding the definitive hosts, need to be considered to explain the varying detection levels observed in the soil and fecal samples in the study areas.

## 5. Conclusions

The current study has clearly demonstrated that both urban and semi-urban areas of the city of Ibadan, Oyo State, Nigeria, are highly contaminated by taeniid, and particularly *E. granulosus s.l.*, eggs. The extent of the environmental contamination found in this study raises public health issues and suggests the adoption of appropriate measures to prevent and control CE. It is recommended that veterinary authorities enforce control over the movement of stray dogs and establish a deworming programme. Finally, parents and caregivers should discourage children from playing with soil in public spaces. Additional studies to molecularly characterise the taeniid species, including *E. granulosus s.l.*, are required to appropriately understand the zoonotic potential of parasitic contamination.

## Figures and Tables

**Table 1:** Environmental factors associated with *E. granulosus s.l.* presence in soil samples (*n* = 200) from open fields in Ibadan.

Variables	Characteristics	*E. granulosus* Positive Soil *n* = 16	*E. granulosus* Negative Soil *n* = 184	Odds Ratio (95% CI)	*p* Value
Distance from abattoir	5 m	0 (0.0)	10 (100.0)	0.00 (0.00, 27.50)	1.00
	10 m	2 (20.0)	8 (80.0)	5.73 (0.37, 89.87)	0.13
	20 m	2 (13.3)	13 (86.7)	3.60 (0.24, 54.13)	0.23
	40 m	1 (5.0)	19 (95.0)	1.26 (0.02, 25.53)	1.00
	100 m	6 (24.0)	19 (76.0)	7.35 (1.18, 80.69)	0.01 *
	200 m	1 (2.9)	34 (97.1)	0.71 (0.01, 14.13)	1.00
	500 m	2 (5.7)	33 (94.3)	1.45 (0.10, 20.91)	1.00
	10 km	2 (4.0)	48 (96.0)	Ref.	
Level of urbanisation	Semi-Urban	2 (8.0)	23 (92.0)	1.00 (0.10, 4.81)	1.00
	Urban	14 (8.0)	161 (92.0)		
Local Government Area	Ibadan North	14 (9.3)	136 (90.7)	Ref.	
	Ibadan South West	0 (0.0)	25 (100.0)	0.00 (0.00, 1.77)	0.23
	Akinyele	2 (8.0)	23 (92.0)	0.85 (0.09, 4.08)	1.00

Values in brackets indicate the percentage of positive and negative samples in each subgroup. * Significant *p* value (*p* ≤ 0.05); CI = Confidence interval.

**Table 2:** Environmental factors associated with *E. granulosus s.l.* presence in fecal samples (*n* = 200) from stray dogs in Ibadan.

Variables	Characteristics	*E. granulosus* Positive Feces *n* = 48	*E. granulosus* Negative Feces *n* = 152	Odds Ratio (95% CI)	*p* Value
Distance from abattoir	5 m	3 (30.0)	7 (70.0)	1.22 (0.18, 6.39)	1.00
	10 m	5 (50.0)	5 (50.0)	2.78 (0.55, 14.39)	0.15
	20 m	3 (20.0)	12 (80.0)	0.72 (0.11, 3.28)	0.74
	40 m	7 (35.0)	13 (65.0)	1.52 (0.42, 5.28)	0.56
	100 m	8 (32.0)	17 (68.0)	1.33 (0.40, 4.28)	0.60
	200 m	7 (20.0)	28 (80.0)	0.71 (0.21, 2.24)	0.61
	500 m	2 (5.7)	33 (94.3)	0.18 (0.02, 0.86)	0.02 *
	10 km	13 (26.0)	37 (74.0)	Ref	
Level of urbanisation	Semi-Urban	6 (24.0)	19 (76.0)	1.00 (0.31, 2.83)	1.00
	Urban	42 (24.0)	133 (76.0)		
Local Government Area	Ibadan North	35 (23.3)	115 (76.7)	Ref.	
	Ibadan South West	7 (28.0)	18 (72.0)	1.28 (0.42, 3.54)	0.62
	Akinyele	6 (24.0)	19 (76.0)	1.04 (0.31, 2.98)	1.00

Values in brackets indicate the percentage of positive and negative samples in each subgroup. * Significant *p* value (*p* ≤ 0.05); CI = Confidence interval.

## Data Availability

Not applicable.
